# Detergent Dissolution Intensification via Energy-Efficient Hydrodynamic Cavitation Reactors

**DOI:** 10.1021/acsomega.3c03517

**Published:** 2023-08-02

**Authors:** Mohammadamin Maleki, Farzad Rokhsar Talabazar, Seyedali Seyedmirzaei Sarraf, Araz Sheibani Aghdam, Songül Bayraktar, Ehsan Tuzcuoğlu, Ali Koşar, Morteza Ghorbani

**Affiliations:** † Faculty of Engineering and Natural Science, Sabanci University, 34956 Tuzla, Istanbul, Turkey; ‡ Sabanci University Nanotechnology Research and Application Center, 34956 Tuzla, Istanbul, Turkey; § R&D Center, Arçelik A.Ş., 34950 Tuzla, Istanbul, Turkey; ∥ Center of Excellence for Functional Surfaces and Interfaces for Nano-Diagnostics (EFSUN), Sabanci University, Orhanli, 34956 Tuzla, Istanbul, Turkey; ⊥ School of Engineering, Computing and Mathematics, Oxford Brookes University, College Cl, Wheatley, Oxford OX33 1HX, U.K.

## Abstract

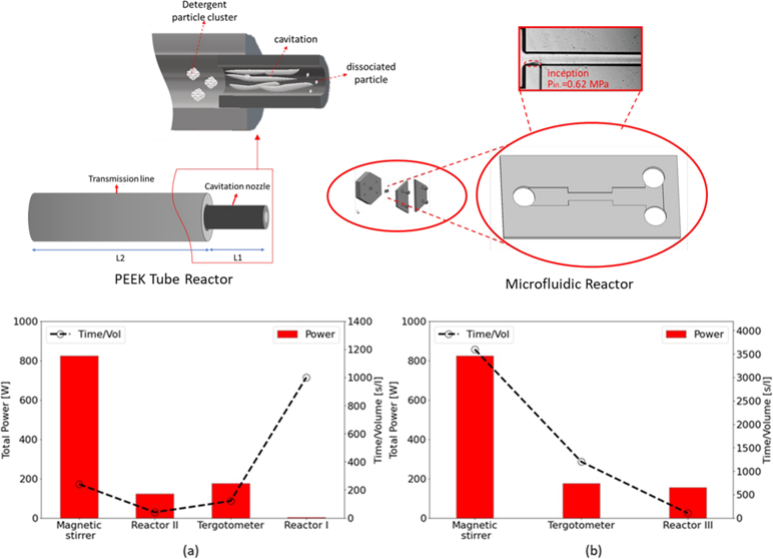

In this study, we explored the potential of hydrodynamic cavitation (HC) for use in dissolution of liquid and powder detergents. For this, microfluidic and polyether ether ketone (PEEK) tube HC reactors with different configurations were employed, and the results from the reactors were compared with a magnetic stirrer, as well as a tergotometer. According to our results PEEK tube HC reactors present the best performance for dissolution of liquid and powder detergents. In the case of liquid detergent, for the same level of initial concentration and comparable final dissolution, the PEEK tube consumed 16.7 and 70% of the energy and time of a tergotometer and 16.7 and 14.8% of that of a magnetic stirrer, respectively. In the case of powder detergent, the PEEK tube used 12% less power than a tergotometer and 81.2% less power than a magnetic stirrer. Additionally, the time required to dissolve the detergent was reduced significantly from 1200 s in the tergotometer and 1800 s in the magnetic stirrer to just 50 s in the PEEK tube. These results suggest that HC could significantly improve the dissolution rate of liquid and powder detergents and energy consumption in washing machines.

## 1 Introduction

The preparation of well-dissolved and homogenous solutions is of great importance in a wide range of scientific and industrial applications such as pharmaceuticals,^1^ food processing,^2^ and wet appliance.^3^ The dissolution process involves the interactions of the solute with the solvent molecules and the motion of the solute molecules into the bulk solution.^4^ For the case of solid particles, the dissolution mechanism typically consists of several steps such as disintegration, disaggregation, and relocation of solute molecules in the solution, while the dissolution occurs directly for the liquid formulation.^1^ The dissolution mechanism is generally controlled by diffusion and/or kinetics, which are affected by major parameters such as temperature, pressure, solute concentration, and solvent composition.^5^

One emerging application of the dissolution process is the preparation of detergent solutions for wet appliances. Improvements in detergent dissolution will lead to higher energy efficiencies of wet appliances, which are among the most widely used household reactors.^6^ Various studies have investigated the effective mechanisms of detergent dissolution. Most of these studies evaluated the internal structures and physicochemical properties of the detergent particles and their influence on dissolution enhancement. For instance, Mutch et al.^7^ have studied the effect of morphological properties of the powder detergent and the granulation method on the detergent dissolution performance. Utilizing a foamed binder, they found that foam granulation resulted in smaller granules with increased surface roughness. They indicated that the mechanical effects of foam granulation, such as enhanced granule structures and narrower particle size distribution, directly influence dissolution rates.^7^ In a separate study, Pan et al.^8^ examined the dissolution mechanisms of detergent agglomerates with various binders. They observed that the dissolution rates of specific detergent components were influenced by the type and content of binders used in the granulation process. Notably, the choice of binder affected the dissolution behavior and mechanisms of detergent agglomerates in water.^8^ Additionally, research by other authors^9,10^ provided insights into the kinetics of detergent dissolution and the effects of various parameters such as temperature, stirring speed, and pH. In a study by Li et al.,^9^ it was found that temperature and stirring speed positively affect the dissolution rate and rate constant of detergent particles, while the pH of the solution negatively influences these parameters. The presence of certain polymers, such as carboxymethyl cellulose was found to enhance the dissolution rate and rate constant of detergent particles. Furthermore, in a numerical study by Cao et al.^10^ the shape, surface area to volume ratio, and pore structure of particles were identified as important physical factors affecting detergent dissolution. Agitation, particularly the shear rate, was found to significantly impact particle dissolution. These findings highlight the importance of considering physical effects on detergent dissolution, which can contribute to the improvement of detergent manufacturing processes. These findings highlight the importance of considering the mechanical and physical effects on detergent dissolution. While foam granulation demonstrated positive properties such as increased surface roughness, these effects did not directly translate into enhanced dissolution rates.

Different mechanisms such as mechanical and pneumatic stirring, evacuation, and cavitation could be exploited to facilitate the dissolution. Among these approaches, cavitation has been emerging as a promising strategy from both energy and time considerations.^11^

Cavitation bubbles are generated due to a reduction in the local static pressure below the saturation vapor pressure. Pressure recovery leads to collapse of the generated bubbles so that a huge amount of energy is released to the surrounding medium.^12^ This energy is characterized by intense mechanical (shock wave and microjet), thermal (local hotspots), and chemical (hydroxyl radicals) effects.^13^ When considering cavitation generation techniques, acoustic cavitation (AC) and hydrodynamic cavitation (HC) are the most popular approaches because of their efficiencies and simple implementations.

In AC, cavitation bubbles are subjected to an acoustic field. Under these circumstances, bubbles experience a rapid contraction–expansion cycle leading to an increase in overall bubble size. When the bubble size reaches a critical value, a sudden collapse of the bubble results in localized hotspots of energy.^14^ This approach is useful due to the controllable nature of a single bubble cavitation.^15^ However, its application on the industrial scale is difficult because of scale up and energy efficiency issues.^16^ In this regard, an efficient alternative approach for industrial-scale applications is HC. In HC, a low local pressure value is achieved when the flow is accelerated within a flow-restrictive element such as an orifice. HC is of great interest in many applications including energy harvesting,^17^ wastewater treatment,^18^ biomedical engineering,^19^ and wet appliances.^3^

There is an increasingly popular application of HC in particle dissolution and disintegration,^5,20,21^ as well as physical and chemical processing.^22,23^ For example, a study by Faraloni et al.^24^ demonstrated the exceptional efficiency of hydrodynamic cavitation (HC) in food processing and material dissociation. The study showed that HC can produce high extraction yields and nutritional profiles comparable to those of a high-end commercial product. Additionally, the use of HC-based processing eliminated the need for multiple time- and energy-consuming pre- and post-treatments, such as soaking, blanching, and peeling. This makes HC a sustainable and efficient alternative for beverage production. One of the initial studies on the effect of HC on particle disintegration was conducted by Dvorsky et al.^20^ In that study, a water jet HC generator was implemented to investigate the impact of cavitation implosion in the dispersion of silicon solid particles. Their results indicated that HC significantly enhanced the disintegration rate of silicon particles. In another study, Vitenko et al.^11^ investigated the effect of HC on the dissolution of kinetically soluble substances (langbeinite particles). According to their study, HC enhanced the mass transfer and fractionation of particles, which in turn facilitated the dissolution of particles. Furthermore, the high destructive capacity of HC has been widely employed in different applications for emulsification, sludge disintegration, particle dissolution, and pollutant degradation.^25−27^ Recently, HC has been proven to be an effective tool for detergent dissolution preparation in household laundry machines.^3,6^ As an example, Perdih et al.^3^ utilized a rotary HC generator to investigate the effect of cavitation on the powder detergent dissolution rate and the textile stain removal performance. In that study, it was shown that cavitation could significantly enhance the powder detergent dissolution rate and could facilitate the textile cleaning process through a mechanism similar to cavitation erosion. In a related study,^6^ the same group investigated the influence of cavitation on the dissolution rate of sodium dodecyl benzene sulfonate (SDBS), a commonly used surfactant in detergent products. Through the use of a high-speed camera, they were able to observe and analyze the dissolution process and its underlying physics. Their results proved that the presence of cavitation significantly enhanced the dissolution rate of the surfactant in water, comparable to the process of cavitation erosion. There is no comprehensive study on the influence of major parameters, such as pressure difference and solute concentration on the liquid/powder detergent dissolution performance in different scales. In this study, we employed a microfluidic reactor as well as a polyether ether ketone (PEEK) tube configuration in milli and conventional scales to evaluate the detergent dissolution performance under the influence of HC for both liquid and powder detergents. We also explored the effect of different parameters such as upstream pressure and solute concentration on the dissolution mechanism. Besides, the influence of detergent concentration on cavitation inception was investigated. Finally, we proved that HC in different scales could significantly enhance the dissolution performance from energy/time perspectives compared to the traditional mixing methods, namely, magnetic stirrer and tergotometer.

## 2 Experimental Setup and Procedure

### 2.1 Materials

OmoColor liquid detergent with a dynamic viscosity of 0.562 Pa·s was obtained and utilized during the liquid detergent experiments.

For the experiments on the powder detergent, the standard powder detergent (detergent A*) was prepared by combining IEC-A^28^ (as the base powder) with bleach components (sodium perborate tetrahydrate and (tetraacetylethylenediamine) TAED), according to the IEC EN 60456 standard. The composition of the standard detergent A* used during our experiments had the following contents: 77% IEC-A + 20% sodium perborate + 3% TAED.

### 2.2 Device Fabrication and Configuration

Two types of HC reactors, namely, microfluidic device and PEEK tube with different scales were utilized in this study to perform the HC-induced dissolution experiments.

#### 2.2.1 Microfluidic Device (Reactor I)

**Figure 1:**
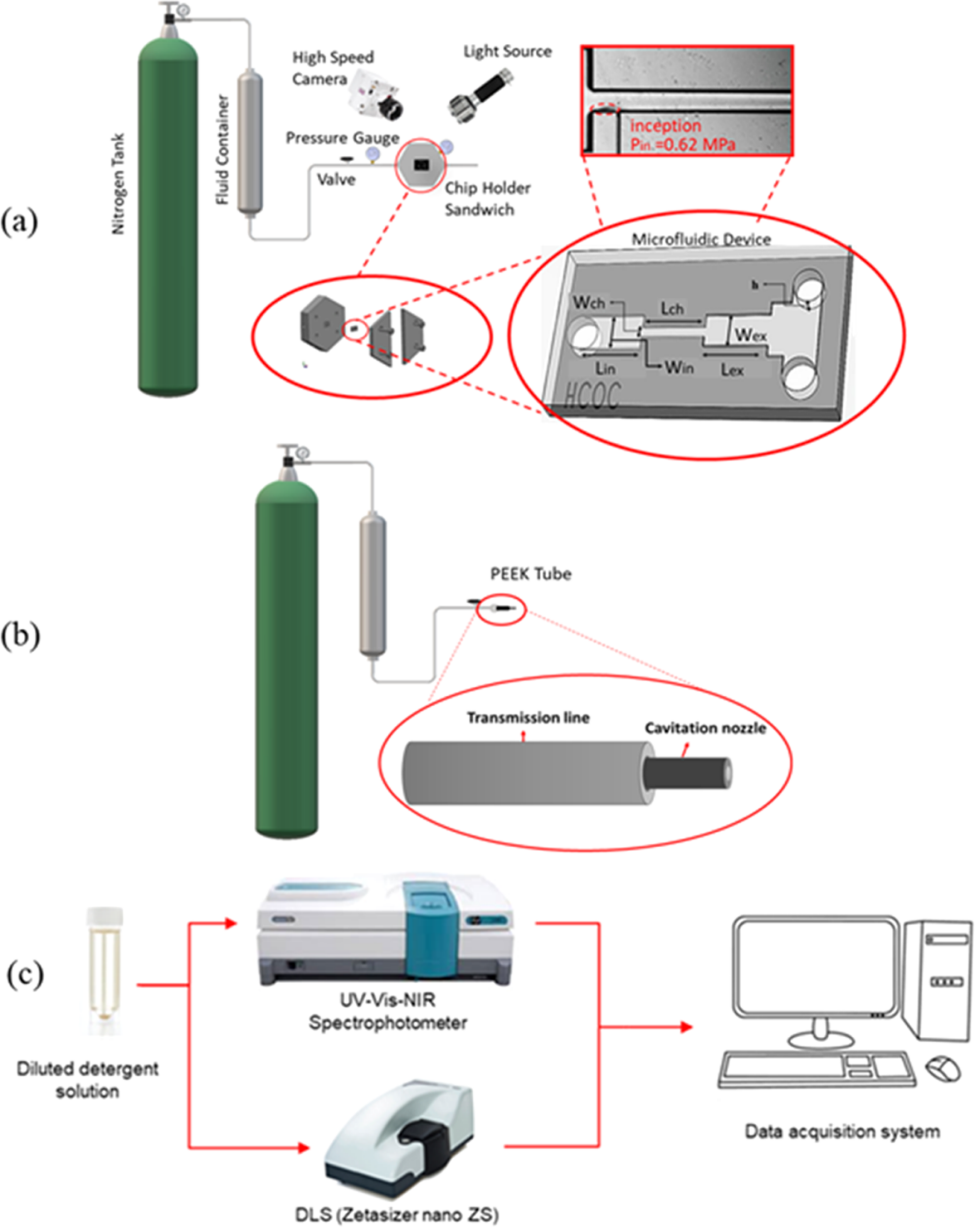
Schematic of the experimental setup for (a) microfluidic device (reactor I), (b) PEEK tube configuration (reactors II and III), and (c) characterization using UV–Vis and DLS devices.

The schematic of the utilized microfluidic device as the HC reactor (reactor I) is shown in Figure 1a. The device was fabricated using standard semiconductor techniques. The silicon-based substrate was bound to Borofloat 33 glass, which allowed for visualization and high-pressure resistance. More details about the fabrication process were provided in our previous studies.^29^ The microfluidic device consists of three main sections including the inlet channel, micro restrictive flow element (microchannel), and extension region. The working conditions of our experiments were determined in such a way that cavitation inception occurred in the microchannel region. The microchannel section had the roughness elements to facilitate cavitation inception and to increase the cavitation intensity.^30^ The details about the device geometry are included in Table 1. The hydraulic diameter calculation used in this study follows the formula:^31^*D*_h_ = 4*A*/*P*, where *A* represents the cross-sectional area and *P* represents the perimeter of the microchannel.

**Table 1:** Microfluidic Device HC Reactor (Reactor I) Configuration

inlet/extension length [μm]	inlet/extension width [μm]	microchannel length (*L*_ch_) [μm]	microchannel width [μm]	channel depth [μm]	microchannel hydraulic diameter (*D*_ch_) [μm]	roughness length [μm]	roughness height [μm]
2000	900	2000	400	50	89	1/3 *L*_ch_	0.1 *D*_ch_

#### 2.2.2 PEEK Tube Configuration (Reactors II and III)

The polyether ether ketone (PEEK) configuration was used as the other HC generator during the experiments. For this, two different PEEK tube sizes (reactors II and III) were utilized in the liquid and powder detergent experiments (Table 2). The main reason for choosing the PEEK tube configuration is that its application in HC generation was examined in our previous studies.^32^ The second reason is that the geometry is very simple and its production is very straightforward. The tube diameters are small enough to demonstrate high-intensity cavitation but large enough to inhibit clotting. In both cases, the small diameter PEEK tube served as the flow-restrictive element for cavitation generation, where a sudden decrease in the cross-section led to a significant pressure drop in the small tube inlet. For each case, the length of the PEEK tube (4.5 mm) was adjusted to have cavitation in the tube outlet region according to our previous study.^23^

**Table 2:** PEEK Tube HC Reactors (II and III) Configuration

	PEEK tube configuration	
type of detergent	inner diameter of transmission line [μm]	inner diameter of cavitation nozzle [μm]
liquid (reactor II)	2032	1016
powder (reactor III)	4500	2032

### 2.3 HC Experimental Test Rig

The open-loop test rig was used for the experiments with the microfluidic reactor (reactor I) and the PEEK tube configuration (reactors II and II). The schematic of the setup is shown in Figure 1. For the case of reactor I, an aluminum sandwich package was designed and utilized to seal and hold the HC reactor. The inlet/outlet and pressure ports of reactor I were sealed with silicon micro-O-rings to prevent leakage. In both cases, the reactors were connected to the water container using stainless-steel tubes (Swagelok). The upstream pressure was supplied using a high-pressure nitrogen tank (Linde Gas, Gebze, Turkey). The pressure was measured using an installed pressure gauge (Omega), and the flow rate was controlled using a valve.

### 2.4 Experimental Procedure

Experiments were performed for different upstream pressures and detergent concentrations. Reactor I was used to investigate the dissolution of the liquid detergent in the microscale, while the PEEK tube HC reactors were utilized for both liquid and powder detergents in milli and conventional scales. In reactor I because of the transparent glass, the cavitation patterns were visible using the visualization system so that the upstream pressure related to cavitation inception pressure can be directly determined. For the PEEK tube configuration, the inception regime was verified with potassium iodide (KI). The details of KI experiments and results are provided in the Supporting Information. In addition, the pressure applied to the solution was set based on the values which were already obtained from the cavitation characterization in our previous study.^33^ The solution was fed to the reactor using the single pass method (the detergent-to-solution ratio ranges from 1 to 30 mL/L, and a constant ratio of 9 mg/L was used for liquid and powder detergent, respectively.) In this method, both water and the detergent are provided in one fluid container before running the experiment, and during the experiment they passed through the same channel. The range of concentrations for the powder and liquid detergents was adopted to simulate the washing conditions according to the ranges provided by Arçelik company, Istanbul, Turkey.

To this end, first, a suitable amount of detergent was poured into the container and then water was added. The solution was not premixed. After applying pressure, the detergent and water passed through the HC reactors, where the solution was exposed to HC. The foam was generated and accumulated on the solution surface after experiments, which was easily separated from the solution surface. 10 mL of samples were taken from the solution immediately after the HC implementation and diluted to be used for characterization. In all experiments, the fluid was passed through the reactor only one time, unless otherwise specified. For the powder detergent, where undissolved detergent particles were noticeable, a 1-h delay was introduced between sampling and characterization to ensure the complete separation and sedimentation of the undissolved components. Subsequently, the homogeneous solution was utilized for dilution and characterization. Dilution was done in a direct manner. In the direct dilution process, there can be some small errors in pipetting. These errors were minimized by multiple sampling (three times) per test.

In addition to the experiments with the open-loop test rig and the HC reactor devices, two mixing approaches were also used to provide a reference for evaluating our results. First, an independent set of experiments were performed in the Research and Development Department of the Arçelik company to replicate the standard home laundry machine conditions. During these experiments, a tergotometer (COPLEY Scientific DC1-300) with an angular velocity of 150 rpm was used for mixing without cavitation. Moreover, a magnetic stirrer (Heidolph Hei-PLATE magnetic stirrers) was used as another dissolution approach, where detergent dissolution was performed with an angular velocity of 150 rpm.

### 2.5 Characterization

Two different characterization techniques were implemented to examine the solubility of the detergents after the experiments. Spectroscopic analysis is the main characterization technique in this study. For this purpose, a UV–Vis spectrophotometer was used to determine the analyte concentration of the obtained solution. In this technique, the number of discrete wavelengths of UV or visible light that were absorbed by or transmitted through a sample in comparison to a reference sample was measured. UV–Vis spectroscopy can be used to determine the solubility of detergents by measuring the absorbance of a detergent solution from the ultraviolet to visible range.^34^ The absorbance spectrum provides insights into the solubility of the detergent because it is directly correlated with the concentration of the absorbing species present in the sample. This relationship is governed by the Beer–Lambert law, which states that absorbance is directly proportional to the concentration of the absorbing species and the path length of the sample.^34^

To determine the extent of detergent dissolution, the tergotometer device was employed, which allowed for precise measurement of the dissolution process. In these tests, the dissolution mechanism was continued until no further change in the peak absorbance was observed, indicating that the detergent had reached its maximum dissolution capacity. The maximum peak absorbance obtained during these tests was considered as representative of complete dissolution. This approach provided a standardized criterion for evaluating the dissolution of the detergent in our study. For the measurement of the mean particle size within the samples, the dynamic light scattering (DLS) technique was employed using a Zetasizer device. The measurement of the scattering light was used to estimate the mean particle size within the sample. DLS measurements were taken three times, and the fluid was tested at least 15 times in each run. The reported z-average represented the particles’ mean hydrodynamic diameter. This measurement was useful to acquire information regarding the influence of the cavitating flow on deagglomeration of the particle clusters. Our recent study^21^ confirmed that cavitating flows could actively deagglomerate nanoparticle clusters.

Average values of experimental measurements were used for the reported velocities and peak absorbance. The measured velocities and the peak absorbance had standard deviations of ±1.5 and ±5%, respectively. Uncertainties in pressure and cavitation number were calculated using the manufacturer’s data sheets and uncertainty propagation method.^24^ Accordingly, the mean uncertainties in pressure, velocity, cavitation number, and peak absorbance are ±1.5, ±0.3, ±4, and ±5%, respectively.

## 3 Results and Discussion

In this study, two different approaches were adopted. Reactors I and II were useful for a controlled study on liquid detergent in micro and milli scales. On the other hand, reactor III was utilized only for powder detergent on the conventional scale. Thus, in this section, first, the dissolution of the liquid detergent in reactors I and II was discussed and in the second part, the powder detergent dissolution in reactor III was scrutinized.

### 3.1 Influence of HC on the Dissolution of Liquid Detergent

Different sets of experiments were performed to investigate the effect of the pressure and concentration on the dissolution rate of the OmoColor liquid detergent. Liquid detergent is a complex mixture composed of several substances, including surfactants, emulsifiers, and various other ingredients. The dissolution process in liquid detergents involves the complete dispersion of the detergent components, such as surfactants and active compounds, and their thorough mixing with the surrounding liquid, typically water, to form a homogeneous solution.^35,36^ In the tests on reactor I, the total volume of the solution (water and the liquid detergent) was 200 mL, while 500 mL of solution was used in the tests on reactor II. All of the experiments were carried out at room temperature (∼20 °C). For UV characterization, the sample with a concentration equal to or smaller than 10 mL/L was diluted with a ratio of 1/40, and the samples with larger concentrations were diluted with a ratio of 1/200. It should be noted that dilution is necessary to have reliable measurements of the peak absorbance, which should be in the range of 0–1.

#### 3.1.1 Upstream Pressure Effect

**Figure 2:**
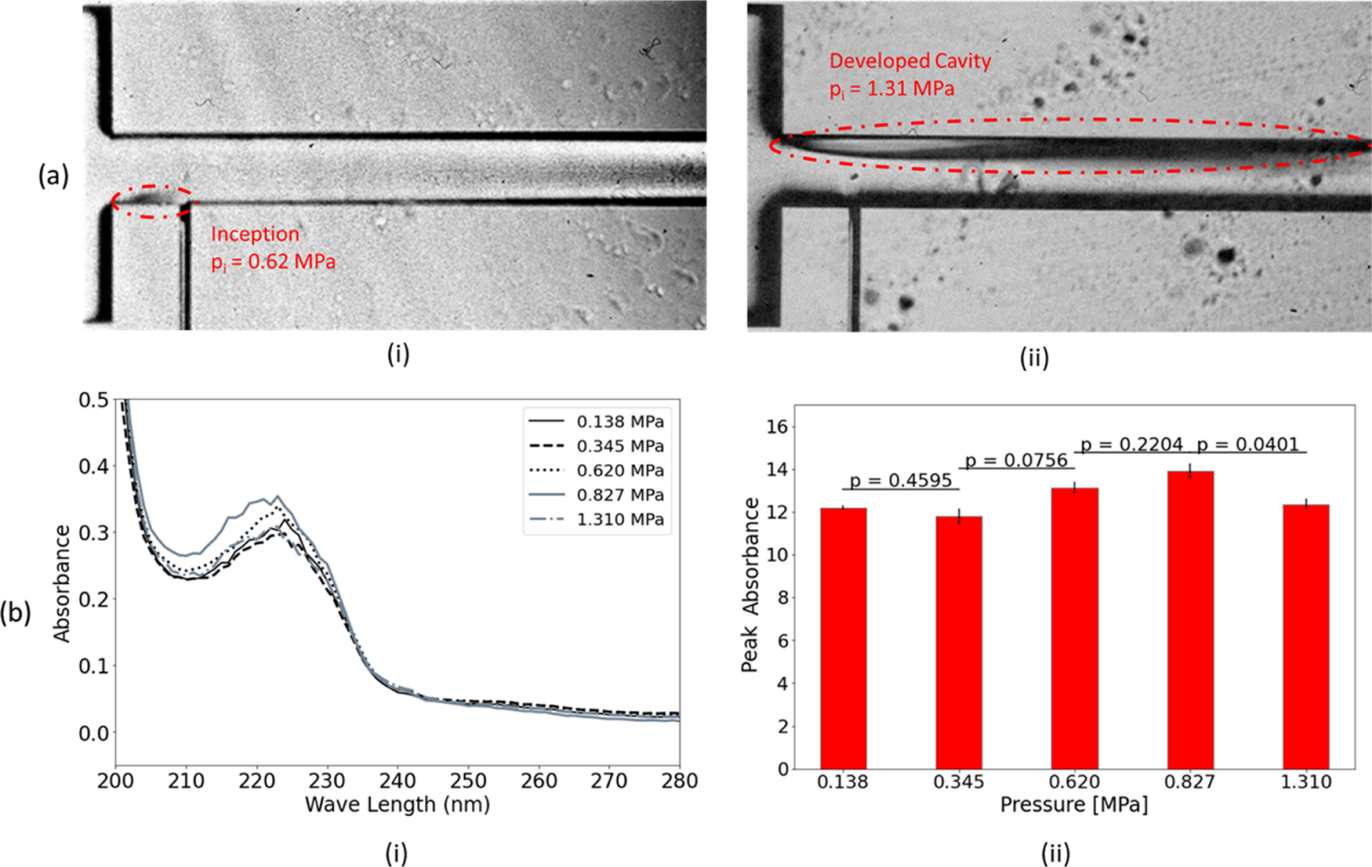
(a) Cavitating flow inside the microchannel (i) inception and (ii) developed cavity and (b) liquid detergent dissolution in reactor I at different upstream pressures (i) UV–vis absorption spectra and (ii) peak absorbance and *p* values of sequential pressures.

In the first set of experiments in reactor I, five different upstream pressures (gauge pressure values) of 0.138, 0.345, 0.620, 0.827, and 1.310 MPa were considered for the 4.5 mL/L ratio of detergent to the solution (Figure 2). The pressures were selected to have different cavitation regimes inside the channel. As a result, the influence of different regimes from no cavitation to developed cavity was investigated. Figure 2b represents the UV measurement results (average of 3 samples for each pressure). The peak absorbance values at a wavelength of 223 nm are shown in Figure 2b(ii). The peak absorbance values represented in all chart bars (including Figure 2b(ii)) are equal to the peak absorbance values obtained from UV–vis characterization multiplied by the dilution coefficient. It can be observed that the peak absorbance decreases by 3.11% with the increase of the pressure from 0.138 to 0.345 MPa, which is due to the decrease in the mass exchange between the solute (detergent) and solvent (distilled water). The dissolution mechanism of miscible liquids depends on fluid and flow properties.^37^ An increase in the upstream pressure augments advection, while the time of the advective mass exchange decreases for a constant volume of the solution. Thus, it can be inferred that the reduction in the advective mass transport time contributes to the decrease in the dissolution of the detergent. The Peclet number determines the dominant mass transport process in this case. Since no information is available in the literature regarding the diffusivity of the detergent, we approximated the Peclet number using the diffusion of a nonionic surfactant (Triton X-100) and considering that surfactants are the main ingredients in detergents. The value provided here serves only as an approximation for determining the dominant mass transport mechanism. According to Weinheimer et al.,^25^ the diffusion coefficient of Triton X-100 at room temperature (25 °C) is in the order of 10^–7^ cm^2^/s. The Peclet number is expressed as,^26^*Pe* = *LU*/*D*, where *L* is the characteristic length, *U* is the local velocity, which was calculated with the use of mass flow rate, and *D* is diffusivity. Based on the flow rate range in the experiments, the Peclet number in this study is in the order of 10^8^, thereby proving significant convective/diffusive mass transport. Other scenarios can contribute to the decrease of peak absorbance as well. One of these scenarios is the feasibility of detergent degradation by cavitation. Previous studies,^38,39^ have demonstrated the effectiveness of hydroxyl radicals in the degradation and oxidation of various substances. An increase in cavity formation can lead to a higher generation of hydroxyl radicals, resulting in a more effective degradation mechanism. UV measurements from the magnetic stirrer and tergotometer tests were obtained and the results showed that peak absorbance occurred in the same wavelengths (220–225 nm) (for more details see Figure 6 and Section 3.3). Since chemicals only absorb very specific wavelengths of light, these results confirm that the main mechanism that leads to the increase in the absorbance should be dissolution.^40^

Upon a further increase of the upstream pressure from 0.345 to 0.620 MPa, despite the reduction in the time of the advective mass transport, a notable jump (11.07% with respect to 0.345 MPa) in the peak absorbance can be recognized. Since a pressure of 0.620 MPa triggers the cavitation inception pressure in reactor I for this solution (Figure 2a(i)), it can be deduced that the cavitation has a positive impact on the dissolution of the liquid detergent.

Cavitation enhances the dissolution in two ways. First, the flow patterns and effects including turbulence and microjets generated in the presence of cavitation result in the enhancement of advective mass exchange of the solute to solvent, and subsequently enhances the dissolution.^41^ Additionally, in a recent study,^21^ it was illustrated that the energy released from the cavitation bubble collapse leads to the deagglomeration of the particle cluster, which further enhances the dissolution rate. As it was shown in our previous study as well^30^ that a further increase in the upstream pressure intensifies cavitating flow while reducing the advective mass transport time. An increase in the upstream pressure from 0.620 to 0.827 MPa further increases the peak absorbance by 5.86%, while a further increase of the upstream pressure (1.310 MPa) reduces the peak absorbance value. The developed cavity for the upstream pressure of 1.310 MPa is observable in Figure 2a(ii). Although it is possible to have supercavitation at high upstream pressures, this would not prevent bubble collapses in the current study. This is because the outlet of the channel is exposed to atmospheric conditions, and according to previous studies^42^ when a stable vapor bubble reaches the outlet and opens to the atmosphere, it will suddenly collapse. This is due to the influence of the bubble opening to the atmospheric condition, which results in a mixture of water vapor and atmospheric air inside the bubble. The behavior of the bubble at this stage is similar to that of an artificial cavity formed by a gas supply.

Furthermore, as the flow velocity decreases, the bubble and pressure remain relatively stable until the bubble collapses, with only a slight change in pressure. This sudden collapse of the bubble near the atmospheric outlet suggests that supercavitation may not always effectively suppress bubble collapses, particularly when the flow velocity decreases significantly in a confined environment such as ducts or microchannels. In this case, because of the high flow rate, the exposure time of the detergent to the cavitation decreases, nonetheless the peak absorbance corresponding to this upstream pressure is still 1% larger than that at 0.138 MPa. In the microchannel shown in Figure 2a, there is an asymmetric cavity associated with the cavitating flow. Similar structures have been observed in other studies,^43,44^ which suggests that they are caused by a combination of factors, including asymmetric inflow/outflow resulting from partial clotting and geometric features such as the pressure port channel along the lower wall.

**Figure 3:**
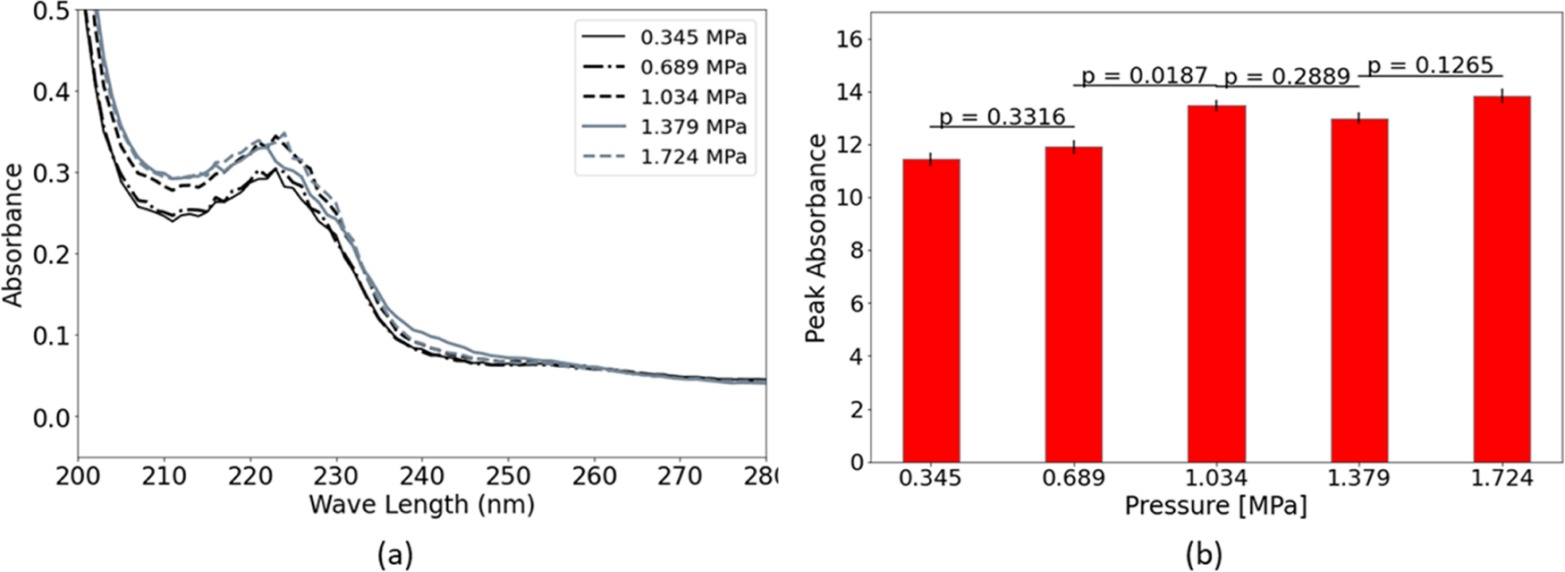
Liquid detergent dissolution in reactor II at different upstream pressures (a) UV–vis absorption spectra, (b) peak absorbance and *p* values of sequential pressures.

The experiments were repeated for reactor II on a milli scale. In this case, upstream pressures of 0.345, 0.689, 1.034, 1.379, and 1.724 MPa were considered. Figure 3 illustrates the UV measurement results, which follow the same trend as the tests in reactor I. This time, a remarkable increase (11.26%) in the peak absorbance can be observed for the upstream pressure of 1.034 MPa, which corresponds to the cavitation inception for reactor II.

To characterize the cavitating flows corresponding to the inception regime for reactor I and reactor II, the cavitation number was used, which is defined as follows^30^
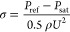
1where σ, *P*_ref_, *P*_sat_, ρ, and *U*, respectively, stand for the cavitation number, reference pressure, saturation vapor pressure (2.33 kPa), fluid density (998.2 kg/m^3^), and velocity in the flow restrictive element. Depending on the placement of measurements and the design of the experiment, various definition for the cavitation number exist in the literature.^45^ In this study, we focus on the cavitation processes observed in an open-loop experimental setup and utilize the upstream pressure (which is measured at the entrance of the cavitation device) as the reference pressure to describe these phenomena. Accordingly, the inception cavitation numbers for reactor I and reactor II are 0.495 and 2.818, respectively. The reason to have a higher cavitation number in reactor II is the larger hydraulic diameter of this reactor compared to reactor I, which leads to a smaller velocity and a higher upstream pressure for the inception.

In most cases, liquid’s temporal motions provide microscopic voids (weak points) that serve as nuclei for liquid rupture and macroscopic bubble formation, which is called homogenous nucleation. Theoretically, when the local pressure within the flow drops to values below the saturation pressure of the working fluid, homogenous nucleation and cavitation occur. Different parameters including the presence of impurities, which influence the working fluid properties such as density and surface tension facilitate or hinder cavitation inception. Besides, particles and impurities can provide heterogeneous nucleation sites to facilitate the inception process.^46^ In the following section, the effect of the detergent concentration on the dissolution and cavitation inception is discussed.

#### 3.1.2 Solute-to-Solution Ratio Effect

**Figure 4:**
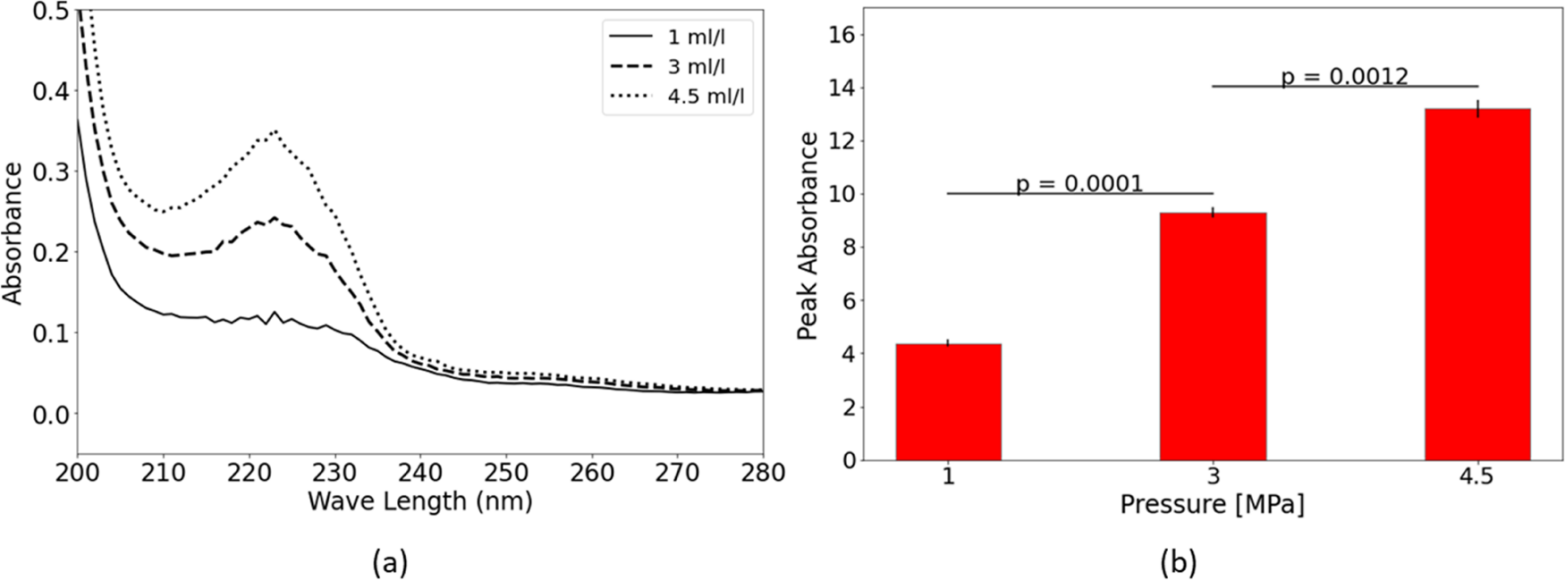
Liquid detergent dissolution in reactor I at different solute concentrations (a) UV–vis absorption spectra and (b) peak absorbance and *p* values of sequential concentrations.

**Figure 5:**
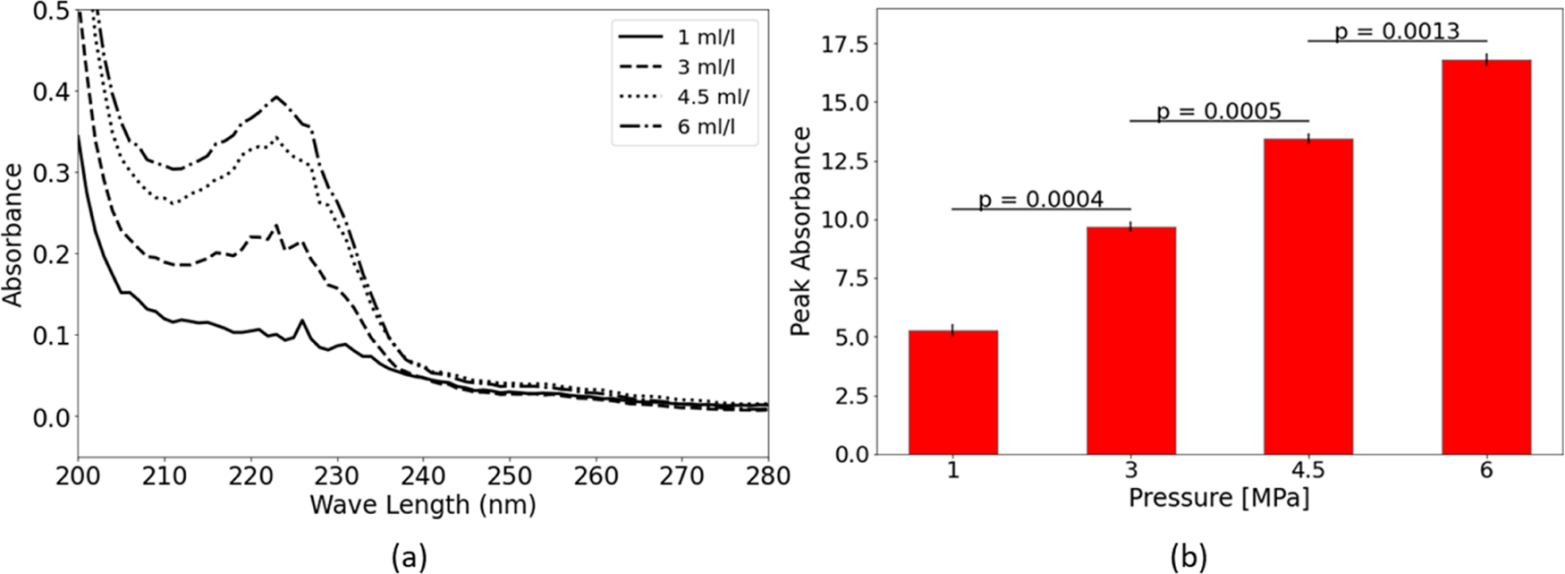
Liquid detergent dissolution in reactor II at different solute concentrations (a) UV–vis absorption spectra and (b) peak absorbance and *p* values of sequential concentrations.

Dissolution results at different volumetric ratios of solute to solution were obtained for both reactors I and II. All of the related experiments were performed at a constant upstream pressure (0.620 MPa for reactor I and 1.340 MPa for reactor II, which corresponds to cavitation inception). As expected, similar trends are observed for reactor I and reactor II tests. Figures 4 and 5 display the UV measurement results for reactor I and reactor II tests, respectively. It can be observed that the peak absorbance values in both cases increase with the solvent-to-solute volumetric ratio.

**Figure 6:**
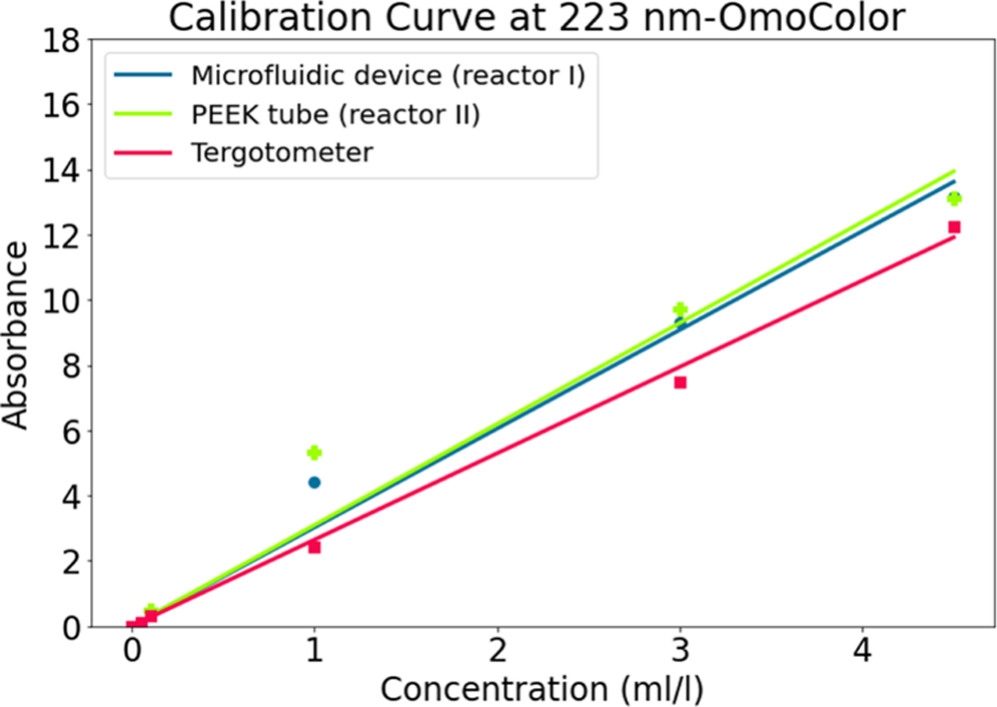
Interpolated calibration curve (peak absorbances) for different experiments with the use of different configurations for OmoColor detergent. The coefficient of determination (*R*^2^) for the calibration curves for reactor I, reactor II, and tergotometer are 0.986, 0.968, and 0.997, respectively.

To have a more comprehensive evaluation of the results, a separate set of tests were done using a standard mixer in the Research and Development Department of the Arcelik company. As a result, the calibration curves for the dissolved liquid detergent were developed using three different methods (reactor I, reactor II, and standard mixer) in Figure 6. The UV measurements were done for the diluted samples. Therefore, in the calibration curves the peak absorbances were multiplied with the dilution ratio. Accordingly, the calibration curves for reactor I and reactor II are very close to each other, which suggests that almost complete dissolution is achieved after passing the detergent through the device and configuration in the cavitating flow regime. Nonetheless, it can be observed that the calibration curve corresponding to tergotometer is different from those of reactor II and reactor I. There can be different sources for this deviation since the experiments using the tergotometer were carried out in a different place the deviation in the calibration curves can have resulted from differences in the batches, sampling, and deviations in dilution. Furthermore, the peak absorbance for reactor I and II at 1 mL/L concentration significantly deviates from the fitted curve. Various sources of error can be associated with the peak absorbance characterization in 1 mL/L concentration. Among these, errors in characterization are considered to be the most probable source. The low solute-to-solvent ratio in this case means that the resulting solution becomes highly diluted after the 1/40 dilution process. As a result, it is plausible that the small errors in the UV–vis device measurement are magnified due to the very low concentration of the diluted solution.

To gain more insight into the effects of the solute-to-solution ratio on cavitation inception, a series of experiments were conducted using the reactor II configuration for three different solutes-to-solution ratios of 4.5, 10, and 30 mL/L and different upstream pressures. The results of these experiments are shown in Table 3. According to these results, the maximum enhancement of the peak absorbance in the cases of 4.5, 10, and 30 mL/L occur at pressures of 1.034, 0.689, and 0.689 MPa. According to the results of reactor I and II in the previous section, cavitation inception is associated with a sudden rise in the PEEK absorbance, suggesting that the same behavior applies to different initial concentrations. Results in Table 3 indicate that for the larger initial concentrations, a sudden rise in the peak absorbance occurs at lower upstream pressures. These results suggest that increasing the initial concentration of detergent leads to a decrease in the inception pressure, the reason for which can be the change in the water tensile strength after mixing with detergent. The obtained results are consistent with previous findings, which show that surfactants (the primary component in detergents) facilitate bubble growth and in consequence inception by influencing surface tension, interfacial resistance to mass transfer, and surface rheological properties.^47,48^ Thus, it can be inferred that the increase in the liquid detergent-to-water ratio leads to cavitation inception in smaller upstream pressures.

**Table 3:** Dissolution of Liquid Detergents Using Reactor II at Different Concentrations and Upstream Pressures

	OmoColor concentration 4.5 [mL/L]					OmoColor concentration 10 [mL/L]			OmoColor concentration 30 [mL/L]		
pressure [MPa]	0.345	0.689	1.034	1.379	1.724	0.345	0.689	1.034	0.345	0.689	1.034
peak absorbance	11.28	11.99	13.34	13.08	13.91	27.98	31.93	30.84	79.99	82.32	82.95

**Figure 7:**
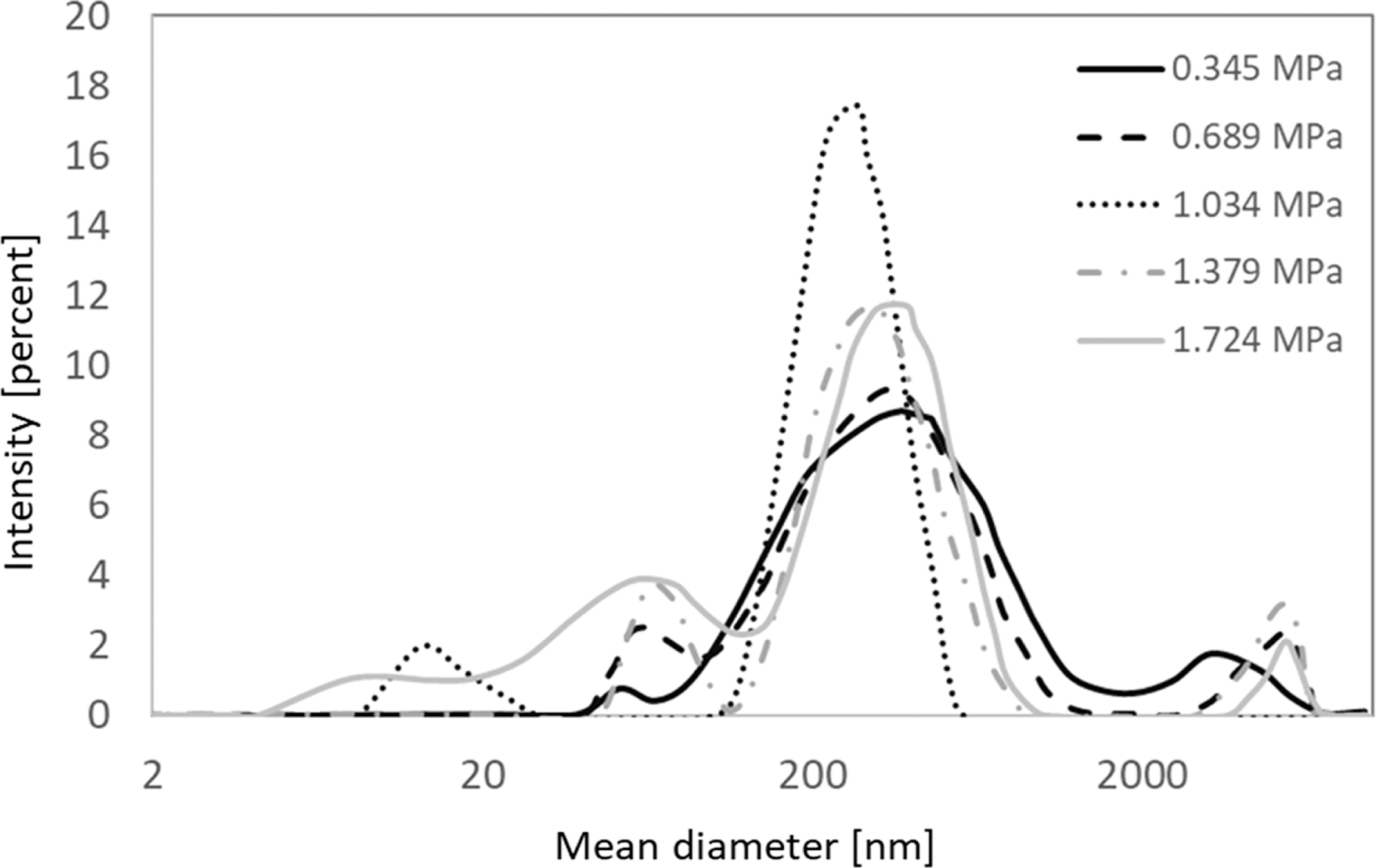
DLS measurement results for reactor II tests at different upstream pressures at the detergent-to-solution ratio of 4.5 mL/L.

Finally, the effect of cavitation on the detergent particles of average hydrodynamic diameter was explored through the DLS measurements (Figure 7 and Table 4). DLS measurements were performed for reactor II tests at a constant detergent-to-solution ratio of 4.5 mL/L and upstream pressures ranging from 0.345 to 1.724 MPa. According to the ζ-average results provided in Table 4, the mean hydrodynamic diameter of the detergent particles is reduced by ∼24% beyond the cavitation inception (upstream pressure of 1.034 MPa). A further increase in the upstream pressure deteriorates the particle deagglomeration, which could be the reason for the significant reduction in the time duration of particle exposure to the bubble collapse. These results confirm the contribution of cavitation to detergent particle deagglomeration and dissolution intensification. One possible scenario in the presence of surfactant is micelle formation by cavitation.^49^ This is particularly the case when there is a high concentration of surfactant in the solution. Nonetheless, no significant evidence for micelle formation was observed in DLS characterization results. According to Figure 7, it is observed that cavitation generation in the channel leads to the reduction in the average size of particles, which indicates that the dominant mechanism in this study should be particle dissociation.

**Table 4:** DLS Measurement Results for Reactor II Tests at Different Upstream Pressures

pressure [MPa]	0.345	0.689	1.034	1.379	1.724
ζ-average [nm]	350.6	349.9	266.0	328.1	334.7

### 3.2 Influence of HC on the Dissolution of Powder Detergent

**Figure 8:**
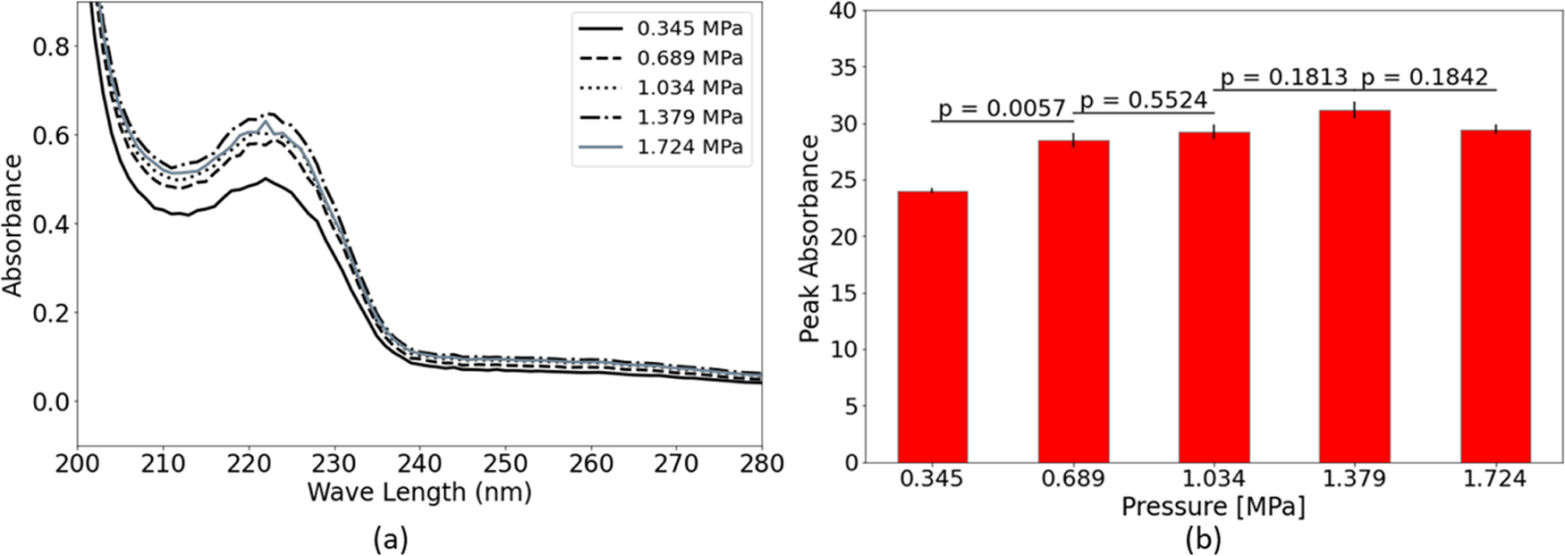
Powder detergent dissolution within reactor III at different upstream pressures (a) UV–vis absorption spectra and (b) peak absorbance and p values of sequential pressures.

The dissolution of the powder detergent within reactor III on a conventional scale was examined at different upstream pressures of 0.345, 0.689, 1.034, 1.379, and 1.724 MPa. All of these experiments were conducted with a standard powder-to-solution ratio of 9 g/L (500 mL solution), and the measurements were made for the dilution ratio of 1/50. The UV measurement results are shown in Figure 8. A remarkable improvement (20.4%) in the peak absorbance can be observed when the upstream pressure is increased from 0.345 to 0.689 MPa. With a further increase in the upstream pressure, the peak absorbance changes at a slower rate (less than 7%). As observed in the case of liquid detergent, cavitation improves the dissolution process. Therefore, it can be concluded that cavitation inception is the main reason for the significant dissolution intensification at a pressure of 0.689 MPa with the corresponding cavitation number of 5.786. Furthermore, the peak absorbance drops after some increase in upstream pressure, consistent with our observations in the case of liquid detergent. As it was mentioned in the previous sections, this reduction in dissolution could be a result of various factors, including a reduction in particle exposure time to cavitation collapse events, or degradation of particles after they are exposed to hydroxyl released during bubble collapses. In addition, similar to the liquid detergent case, the presence of the powder particles in the solution positively affects the cavitation inception process, which is in line with the concept of heterogeneous nucleation in the presence of solid particles in the working fluid.

**Figure 9:**
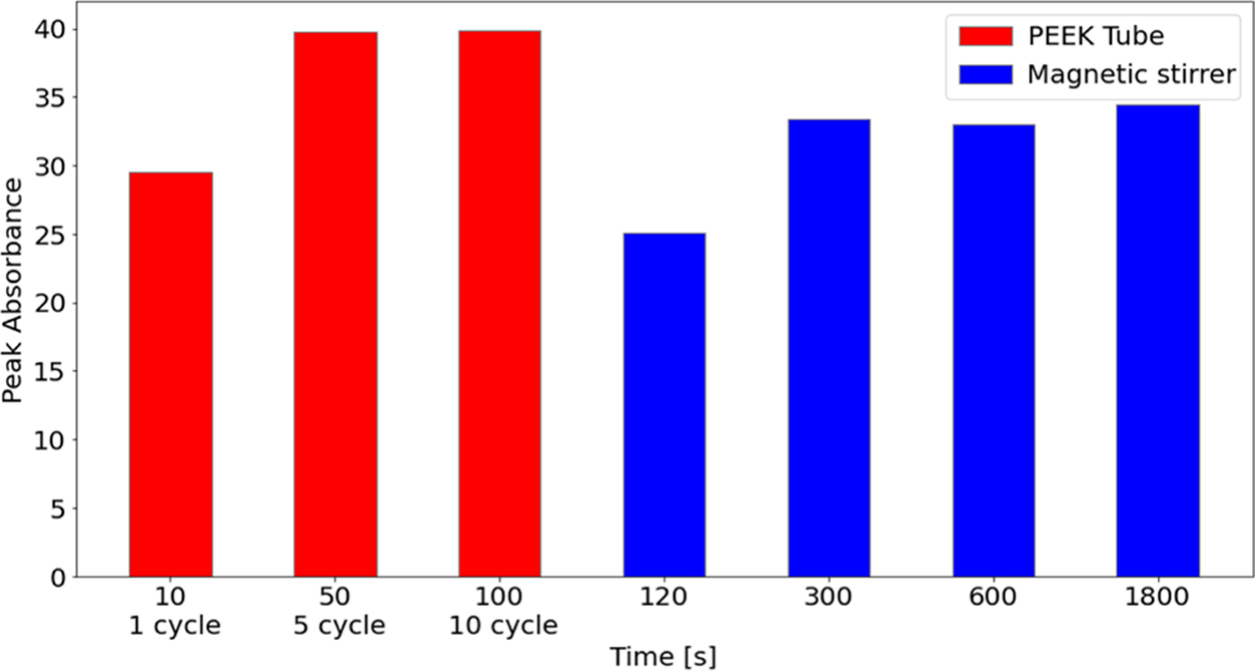
Comparison of powder detergent dissolution at different time durations for reactor III (PEEK tube) and magnetic stirrer cases.

In addition, we employed a magnetic stirrer for a more comprehensive evaluation of the cavitation effect on the dissolution rate. In this case, a 500 mL solution with a detergent-to-solution ratio of 9 g/L was prepared and mixed using the magnetic stirrer with an angular velocity of 150 rpm. The UV measurement results for different mixing times are compared with the results of reactor III tests for a different number of test cycles (1, 5, and 10 cycles) and at the upstream pressure of 0.689 MPa. Figure 9 depicts the peak absorbance values obtained from the samples during these experiments. It can be observed that for the reactor III case, the peak absorbance value is remarkably enhanced by 34.9% with an increase in the number of cycles from 1 to 5. A further increase in the number of cycles has no considerable influence on the peak absorbance value. For the mixer case, the peak absorbance increases with time. However, the increase in the peak absorbance for the duration between 300 and 1800 s is less than 3.3%, which suggests that a further increase in duration has a negligible influence on the dissolution of the powder detergent. A comparison among results leads to the conclusion that the maximum peak absorbance obtained from reactor III after 5 cycles is 15.4% larger than that of the mixer after 1800 s of mixing. In our previous study,^21^ it was shown that HC increased particle cluster deagglomeration, which facilitated the dissolution rate. From these results, it can be inferred that cavitation directly improves the saturation solubility of the powder detergent through particle cluster deagglomeration, which could be otherwise possible only by increasing the sample temperature through heating. Therefore, cavitation serves as an alternative to heating for intensification of the powder detergent solubility in related applications such as wet appliances.

### 3.3 Power Consumption Analysis

**Figure 10:**
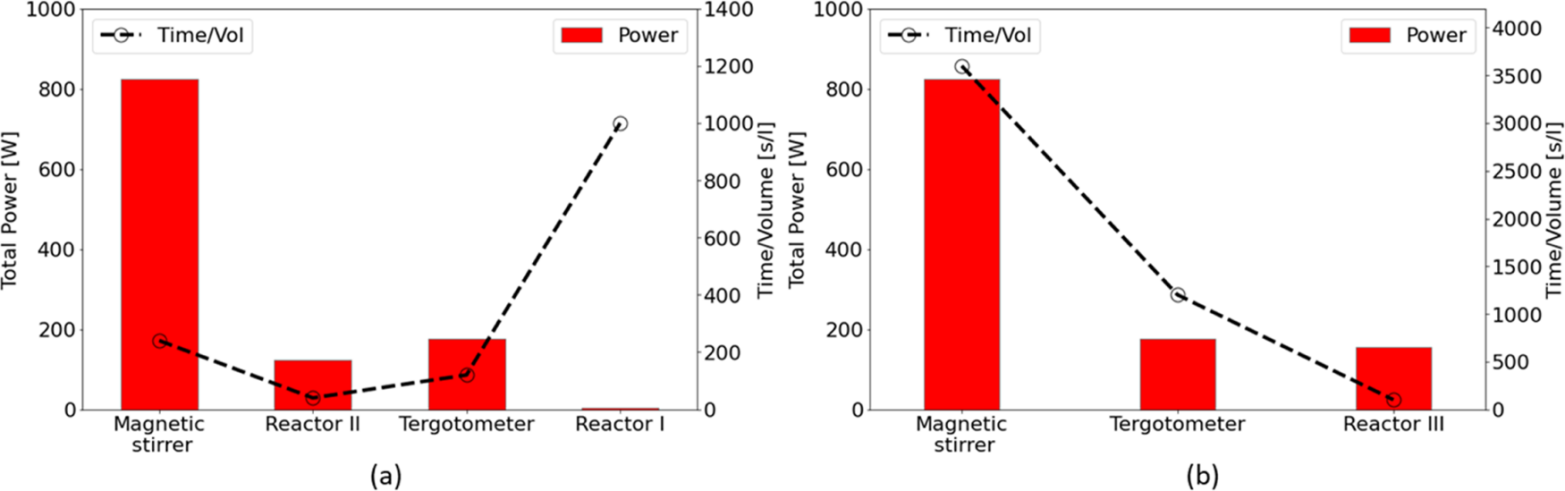
Power and time consumption for unit volume of solution in different devices for (a) liquid detergent and (b) powder detergent.

The systems used for dissolution of detergents in distilled water are evaluated based on the energy and time needed to achieve the maximum solubility. The results from the tests with a detergent-to-solution ratio of 4.5 mL/L (Figure 7) and 9 g of detergent per L of solution (Figure 10) were used to evaluate the dissolution of liquid detergent and powder detergent, respectively. Moreover, UV-spectroscopy measurements are considered for evaluation of dissolution. For the cases of reactors I, II, and III, energy consumption is estimated based on the required energy to provide the necessary upstream pressure by a pump. Accordingly, the input power into the liquid is given as^50^
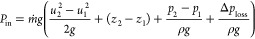
2where *P*_in_ is the input power to the liquid, *ṁ* is the mass flow rate, *g* is the gravitational constant, *z* is the geodetic height, *u* is the velocity, and Δ*p*_loss_ is the power loss. The term in parenthesis stands for the liquid head (*H*). Indices 1 and 2 stand for atmospheric and upstream conditions. Regarding the input power estimation, *u*_2_ = *u*_1_, *z*_2_ = *z*_1_, and the power losses are ignored. To estimate the total power consumption using the liquid head and flow rate, the DPH(S)I pump model was considered.^51^ The total power consumption is the actual power consumption of the mentioned pump model based on the range of the input power consumption and the related flow rate. In performance analysis, only total power consumption is used for comparison between different reactors. Moreover, the total energy consumed by the magnetic stirrer was obtained using the device datasheet (the power is 825 W).^52^ The results corresponding to the consumed time and power for the liquid and powder detergents are listed in Tables 5 and 6. Moreover, Figure 10 illustrates the total power and time per unit volume for achieving the maximum solubility for each case. It should be noted that the maximum solubility achieved by each case can be different. In the case of liquid detergent, the maximum solubility obtained from UV-spectroscopy measurements is very close across all methods, with a maximum difference in peak absorbance of only 6.7% (Figure 7). In contrast, the maximum difference in peak absorbance for powder detergent is more significant, with the magnetic stirrer achieving a solubility that is 15.4% smaller than that of reactor II. As discussed in Section 3.2, this difference is likely due to the dissociation of powder particles by cavitation in reactor II, which remarkably improves the dissolution performance. The test conditions for the magnetic stirrer and tergotometer are described in Section 2.4. According to Figure 10a, it can be observed that for the liquid detergent the reactor II configuration has the best performance in time. Reactor I has the minimum power consumption; however, the operation time of this device is too long, which results in relatively large energy consumption. Therefore, the total energy per unit volume used by both reactors is in the same order (3200 J/L for reactor I vs 4900 J/L for reactor II), while the mixing time for reactor I is much longer (1000 s/L for reactor I vs 40 s/L for reactor II). Another important point is that the time consumption per unit volume for the case of reactor II can be easily diminished by implementing multiple reactors in a parallel arrangement. This allows the flow rate to be increased several times while maintaining approximately the same power consumption per volume. Furthermore, in the case of the powder detergent, it is clear that reactor III is significantly more efficient in time and energy consumption compared to the magnetic stirrer. The power consumption of reactor III is comparable to that of the tergotometer. Despite this, its mixing time and total energy consumption per unit volume of solution are ∼91.7 and 92.7% smaller, respectively, than those of a tergotometer. Considering the cost of electricity as USD 0.05/kWh,^53^ the economic estimation of 1 L of liquid/powder detergent solution preparation using different configurations are provided in Tables 5 and 6. These results highlight the remarkable potential of the suggested HC generators in the intensification of the dissolution while reducing cost and environmental impacts.

**Table 5:** Time and Energy Consumption Evaluation for Different Dissolution Approaches (Liquid Detergent)

parameters	microfluidic device (reactor I), *p*_ref_ = 0.62 MPa	PEEK tube configuration (reactor II), *p*_ref_ = 1.034 MPa	magnetic stirrer	tergotometer
time (s)	200	20	120	120
input power (W)	0.62	25.85		
total power (W)	3.2	122.5	825	176
estimated energy cost (USD/L)	4.44 × 10^–5^	6.8 × 10^–5^	275 × 10^–5^	29.33 × 10^–5^

**Table 6:** Time and Energy Consumption Evaluation for Different Dissolution Approaches (Powder Detergent)

parameters	PEEK tube configuration (reactor III), *p*_ref_ = 0.689 MPa	magnetic stirrer	tergotometer
time (s)	50	1800	1200
input power (W)	34.45		
total power (W)	155	825	176
estimated energy cost (USD/L)	2.15 × 10^–4^	412.5 × 10^–4^	29.33 × 10^–4^

## 4 Conclusions

This study investigated the effect of hydrodynamic cavitation (HC) on the dissolution of liquid and powder detergents. Two HC generators, a microfluidic device (reactor I), and a PEEK tube configuration (reactors II and III), were used to enhance dissolution. Cavitation significantly improved the dissolution of both liquid and powder detergents compared to a tergotometer and a conventional magnetic stirrer. The concentration of the detergent solution notably increased upon cavitation inception, demonstrating the role of cavitation in intensifying dissolution rates. The solubility of powder detergent was enhanced by cavitation-induced deagglomeration, surpassing the solubility increase achievable through temperature elevation. Liquid detergent dissolution showed that a higher concentration facilitated cavitation inception. The proposed HC generators exhibited improved time and energy efficiency compared to conventional mixers. Among all of the devices examined, the PEEK tube configuration exhibited the highest level of efficiency. It is worth noting that the HC reactors have great potential for scalability,^54^ and there is a feasibility of achieving even greater efficiency gains through the expansion and parallel operation of multiple PEEK tube reactors. These findings highlight the potential of integrating HC generators into household laundry machines for enhanced dissolution and reduced energy consumption.

## Supporting Information Available

Details of KI experiments and results with HC reactor II at different upstream pressures, including UV–vis absorption spectra of KI solution (PDF)
