# Supplementary material for: Detergent Dissolution Intensification via Energy-Efficient Hydrodynamic Cavitation Reactors
Source: ACS Omega. 2023 Aug 2;8(32):29595–607. doi: 10.1021/acsomega.3c03517 (PMC10433497; doi:10.1021/acsomega.3c03517)
Supplement: Supplementary file 1 — ao3c03517_si_001.pdf [file ao3c03517_si_001.pdf]

Supplementary Material:

Detergent Dissolution Intensification via Energy-Efficient Hydrodynamic Cavitation Reactors

Mohammadamin Maleki[1,2], Farzad Rokhsar Talabazar[1,2], Seyedali Seyedmirzaei Sarraf[1,2], Araz Sheibani Aghdam[1,2], Songül Bayraktar[3], Ehsan Tuzcuoğlu[3], Ali Koşar[1,2,4,*], Morteza Ghorbani[1,2,4,5,*]

[1]Faculty of Engineering and Natural Science, Sabanci University, 34956 Tuzla, Istanbul, Turkey

[2]Sabanci University Nanotechnology Research and Application Center, 34956 Tuzla, Istanbul, Turkey

[3]Arçelik A.Ş., R &D Center, 34950 Tuzla, Istanbul, Turkey

[4]Center of Excellence for Functional Surfaces and Interfaces for Nano-Diagnostics (EFSUN), Sabanci University, Orhanli, 34956, Tuzla, Istanbul, Turkey

[5]School of Engineering, Computing and Mathematics, Oxford Brookes University, College Cl, Wheatley, Oxford, OX33 1HX, UK

[*] Correspondence authors: Morteza Ghorbani (mghorbani@sabanciuniv.edu) and Ali Koşar (kosara@sabanciuniv.edu)

SMI1. HC characterization in PEEK tube reactor:

Potassium Iodide (KI) solution was exploited to investigate the cavitation regime inside the PEEK tube reactor (reactor II) based on the ion oxidation. The local extreme energy release associated with bubble collapse in the cavitating flow leads to dissociation of H_2O to form OH radicals[1]. Then, in a series of intermediate reactions, the radicals react with the iodide ion (I^-) to form the triiodide complex. The details of the related chemical reactions are provided

in a recent study by our group[2], in which it was shown that the concentration of I^- ions in the solution is minimum in the inception regime, while it is maximum before in small upstream pressures before the inception happens. The concentration of ion was estimated based on peak absorbance of the UV–Vis spectroscopy at wave length around 353 nm. Here, the same methodology was employed to characterize cavitation regime inside the reactor II in different upstream pressures. Thus, three different experiments were carried out with 0.345, 1.034, and 1.724 MPa upstream pressures, and 166 g/L KI solution in deionized water for each experiment. The results for the UV-Vis measurements are shown in Figure S1. The results confirm that the upstream pressure of the 1.034 MPa is associated with the cavitation inception mechanism.

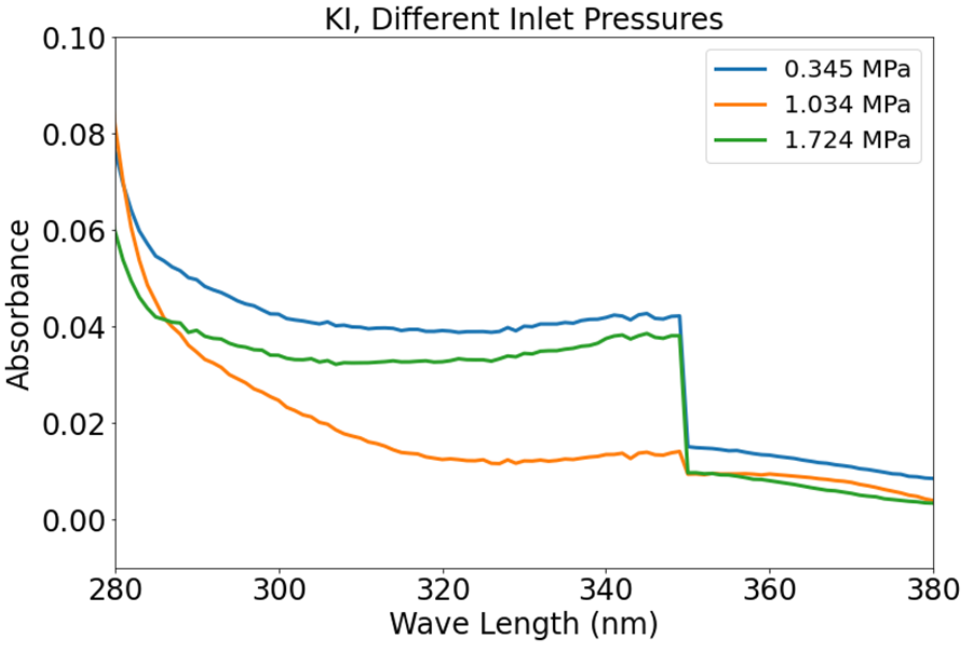

Figure S1 UV-vis absorption spectra of KI solution after experiments with reactor II. The peak absorbance values for upstream pressures of 0.345, 1.034, and 1.742 MPa are 0.0422, 0.0141, and 0.0381 respectively.

References:

[1] K.R. Morison, C.A. Hutchinson, Limitations of the Weissler reaction as a model reaction for

measuring the efficiency of hydrodynamic cavitation, Ultrason Sonochem. 16 (2009) 176–183. https://doi.org/https://doi.org/10.1016/j.ultsonch.2008.07.001.

[2] F. Rokhsar Talabazar, A. Sheibani Aghdam, M. Jafarpour, D. Grishenkov, A. Koşar, M. Ghorbani, Chemical effects in "hydrodynamic cavitation on a chip": The role of cavitating flow patterns, Chemical Engineering Journal. 445 (2022) 136734. https://doi.org/https://doi.org/10.1016/j.cej.2022.136734.
